# Feedback Inhibition in the PhoQ/PhoP Signaling System by a Membrane Peptide

**DOI:** 10.1371/journal.pgen.1000788

**Published:** 2009-12-24

**Authors:** Andrew M. Lippa, Mark Goulian

**Affiliations:** 1 Cell and Molecular Biology Graduate Group, University of Pennsylvania School of Medicine, Philadelphia, Pennsylvania, United States of America; 2 Department of Biology, University of Pennsylvania, Philadelphia, Pennsylvania, United States of America; Stanford University, United States of America

## Abstract

The PhoQ/PhoP signaling system responds to low magnesium and the presence of certain cationic antimicrobial peptides. It regulates genes important for growth under these conditions, as well as additional genes important for virulence in many gram-negative pathogens. PhoQ is a sensor kinase that phosphorylates and activates the transcription factor PhoP. Since feedback inhibition is a common theme in stress-response circuits, we hypothesized that some members of the PhoP regulon may play such a role in the PhoQ/PhoP pathway. We therefore screened for PhoP-regulated genes that mediate feedback in this system. We found that deletion of *mgrB* (*yobG*), which encodes a 47 amino acid peptide, results in a potent increase in PhoP-regulated transcription. In addition, over-expression of *mgrB* decreased transcription at both high and low concentrations of magnesium. Localization and bacterial two-hybrid studies suggest that MgrB resides in the inner-membrane and interacts directly with PhoQ. We further show that MgrB homologs from *Salmonella typhimurium* and *Yersinia pestis* also repress PhoP-regulated transcription in these organisms. In cell regulatory circuits, feedback has been associated with modulating the induction kinetics and/or the cell-to-cell variability in response to stimulus. Interestingly, we found that elimination of MgrB-mediated feedback did not have a significant effect on the kinetics of reporter protein production and did not decrease the variability in expression among cells. Our results indicate MgrB is a broadly conserved membrane peptide that is a critical mediator of negative feedback in the PhoQ/PhoP circuit. This new regulator may function as a point of control that integrates additional input signals to modulate the activity of this important signaling system.

## Author Summary

The proteins PhoQ and PhoP comprise an environmental sensing system that has been extensively studied in numerous bacteria, including *Salmonella typhimurium* and *Escherichia coli*. The PhoQ/PhoP system is stimulated by conditions of low extracellular magnesium or the presence of certain cationic antimicrobial peptides; and it controls genes, whose protein products protect the cell under these conditions or play other critical roles in regulating the virulence of pathogens. The functions of many members of the PhoP regulon, however, remain uncharacterized. This leaves open the possibility that some PhoP-regulated genes may mediate feedback in this system. Regulatory circuits that allow adaptation to environmental change often make use of negative feedback to achieve the appropriate level of response. To look for negative feedback, we screened knockouts of PhoP-regulated genes in *E. coli*. We have identified a remarkably small membrane protein of just 47 amino acids that mediates potent negative feedback on the PhoQ/PhoP circuit in *E. coli*, *S. typhimurium*, *Yersinia pestis*, and likely other related bacteria. This represents a striking example of a small, easily-overlooked open reading frame that plays a critical role in regulating a broadly conserved signal transduction pathway.

## Introduction

Many prokaryotes inhabit multiple niches with disparate environmental conditions and challenges for proliferation. Not surprisingly, they have evolved a plethora of regulatory circuits that enable them to adapt to these environments. One important and extensively studied example is the signaling system controlled by the sensor kinase PhoQ and response regulator PhoP, which is found in enterics such as *Escherichia coli*, *Salmonella typhimurium*, and related bacteria. This two-component system is activated by signals such as low Mg^2+^ [1], low pH [2], or the presence of antimicrobial peptides [3], and leads to expression of genes that encode, among others, Mg^2+^ transporters, enzymes that modify the cell envelope and confer resistance to cationic antimicrobial peptides [4]–[8], enzymes that alleviate stress associated with low pH [9]–[11], and other factors that regulate virulence in numerous gram-negative pathogens of humans, insects, and plants [12]–[18].

The sensor kinase PhoQ is an integral membrane protein whose periplasmic domain is involved in signal detection. Signal transduction occurs via PhoQ autophosphorylation, phosphotransfer to PhoP, and PhoQ-mediated dephosphorylation of phospho-PhoP. Magnesium sensing appears to be mediated by an acidic patch in the periplasmic domain of PhoQ, which structural data suggests is proximal to the membrane [19]–[22]. This acid pocket may also play a role in antimicrobial peptide sensing via displacement of divalent cations by these positively charged peptides [3].

In many cases, adaptive regulatory systems have some form of negative feedback to modulate the cellular response. Indeed, negative feedback is a common theme in cell regulation and its role in maintaining homeostatic control is well-established [23],[24]. Negative feedback can also play a similar role in reducing cell-to-cell variability within a population [25] and can increase the activation kinetics in some circuits [26],[27]. More generally, negative feedback, when combined with additional layers of regulation, may produce complex dynamics or process multiple input signals.

Since the PhoQ/PhoP system functions as a critical stress response circuit for survival under conditions of low magnesium or in the presence of antimicrobial peptides, we hypothesized that there may be sources of negative feedback in this circuit. We would expect that such a negative feedback loop would have at least one component that is regulated by PhoP. Microarray and sequence analysis indicate PhoP influences transcription of a large set of genes [11],[28],[29]. However, relatively few genes have been shown to be directly regulated by PhoP [11],[28],[30], and few of these have known functions. From this short list, none appeared to be obvious candidates for mediators of negative feedback. We therefore screened a reporter strain containing deletions of different PhoP-regulated genes for evidence of increased PhoQ/PhoP-dependent transcription. From an analysis of seven deletion strains, we found that *mgrB* (*yobG*), which is predicted to encode a 47 amino acid peptide of unknown function, plays a critical role in regulating the PhoQ/PhoP pathway.

## Results

### Modulation of PhoP-regulated transcription by MgrB

**Figure 1:**
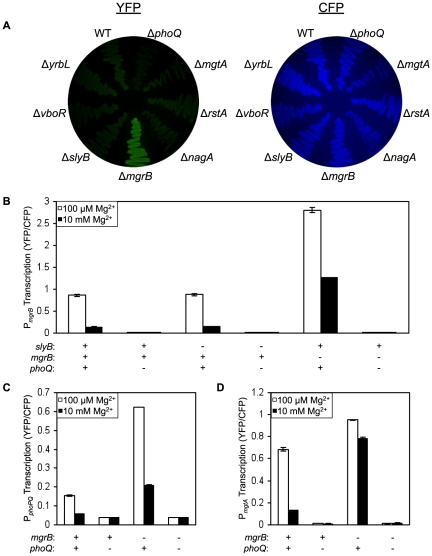
Deletion of *mgrB* leads to up-regulation of multiple genes in the PhoQ/PhoP Regulon. (A) YFP (left) and CFP (right) fluorescence images of a transcriptional reporter strain with deletions of various PhoP-regulated genes, growing on LB agar. The strain contains a chromosomal copy of the PhoP-regulated *mgrB* promoter (P*_mgrB_*) driving *yfp* expression and a constitutive promoter driving *cfp* expression as an internal reference. The specific strains are, clockwise starting with WT, TIM92, TIM100, AML4, AML6, AML8, AML16, AML10, AML12, and AML14. (B) Fluorescence of several strains (as in A) grown in liquid. (C) Fluorescence of *phoPQ* transcriptional reporters grown in liquid. The strains are, from left to right, TIM148, TIM229, AML22, and AML23. (D) Fluorescence of *mgtA* transcriptional reporters grown in liquid. The strains are, from left to right TIM91, TIM99, AML24, AML25. For liquid cultures, cells were grown in minimal glucose medium with 100 µM (white) or 10 mM (black) MgSO_4_ and cellular fluorescence was measured by microscopy and image analysis as described in Materials and Methods. The error bars represent the range of means for two independent cultures.

**Figure 2:**
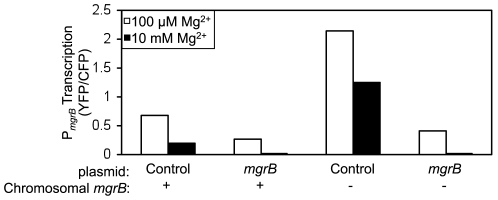
Expression of *mgrB* from a plasmid complements a chromosomal deletion. Reporter strains for *mgrB* transcription are either wild-type for the chromosomal *mgrB* (TIM92) or contain a deletion (AML20) and either a control plasmid (pEB52) or a plasmid bearing *mgrB* (pAL8). Cells were grown in minimal glucose medium with 100 µM (white) or 10 mM (black) MgSO_4_. Cellular fluorescence was measured by microscopy and image analysis as described in Materials and Methods. The error bars represent the range of means for two independent cultures.

To identify factors that mediate negative feedback in the PhoQ/PhoP circuit, a set of genes that have been shown to be directly regulated by PhoP in *E. coli* [28],[30] were individually deleted in a PhoP transcriptional reporter strain. The reporter contained the PhoP-regulated *mgrB* promoter driving YFP expression (P*_mgrB_-yfp*), which was integrated at the phage lambda attachment site. The strains also expressed CFP from a constitutive promoter, which served as an internal control for protein expression and fluorescence intensity. We chose the *mgrB* transcriptional reporter because it gives the highest level of fluorescence from among a collection of fluorescent reporters of PhoP-regulated transcription [31]. As seen in cells growing on LB agar plates, which are moderate inducing conditions for the *E. coli* PhoQ/PhoP system [30], deletion of *mgtA*, *rstA*, *nagA*, *slyB*, *vboR*, and *yrbL* had no visible effect on YFP fluorescence (Figure 1A). Deletion of *mgrB*, however, resulted in a clear increase in YFP with no visible difference in CFP fluorescence when compared with the wild-type strain (Figure 1A). To obtain a quantitative measure of the effects of the gene deletions on transcription levels, we measured the ratio of YFP to CFP fluorescence in single cells by fluorescence microscopy and image analysis [32]. Cells were grown in medium containing either 100 µM or 10 mM Mg^2+^. Consistent with the results observed on agar plates, deletions of *mgtA*, *rstA*, *nagA*, *slyB*, *vboR*, and *yrbL* showed no effect on the YFP/CFP fluorescence (the example of *slyB* is shown in Figure 1B, other examples are shown in Figure S1). Deletion of *mgrB*, on the other hand, resulted in roughly a 9-fold increase in YFP/CFP fluorescence at 10 mM Mg^2+^ and a 3-fold increase at 100 µM Mg^2+^, relative to the corresponding wild-type strain (Figure 1B). The observed increase in fluorescence was strictly dependent on PhoQ (Figure 1B). Deletion of *mgrB* in fluorescent transcriptional reporter strains for three other PhoP-regulated promoters, P*_mgtA_*, P*_phoPQ_*, and P*_hemL_*, resulted in similar increases in fluorescence (Figure 1C and 1D, and data not shown), suggesting that the effect of the deletion is likely to be at a point upstream in the pathway common to all of these genes, i.e. at some point in PhoQ/PhoP signaling. We also verified that the *mgrB* deletion could be complemented by a plasmid containing *mgrB* cloned downstream of the *trc* promoter (Figure 2). When this same plasmid was put in an *mgrB* ^+^ strain, it resulted in a further repression of reporter gene transcription (Figure 2), presumably due to increased expression of MgrB above the wild-type level. Taken together, the above results suggest that the *mgrB* gene product acts as a repressive factor in PhoQ/PhoP signaling.

### MgrB localization

**Figure 3:**
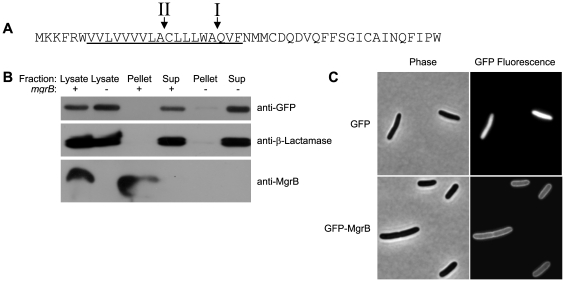
MgrB localizes to the inner membrane *in vivo*. (A) The amino acid sequence of MgrB. The underlined region is a potential transmembrane domain [36]. The arrows labeled I and II denote potential type I ([34] ) and type II [35] signal sequence cleavage sites, respectively. (B) Western blot of the total cell lysate, envelope fraction (pellet), or the soluble protein fraction (sup), of an *mgrB^−^* strain expressing cytoplasmic YFP and CFP (AML20) and containing either a control plasmid (pEB52) or an *mgrB* expression plasmid (pAL8). Both plasmids also express beta-lactamase, an enzyme that resides in the periplasm. (C) Phase contrast and GFP fluorescence micrographs of AML67 (*mgrB* ^−^) expressing either GFP (pAL39, top) or a fusion of GFP to the N-terminus of MgrB (pAL38, bottom). Cells were grown in minimal glucose medium with 1 mM MgSO_4_.

The *mgrB* gene consists of a small open reading frame of 141 base pairs. A recent study of small open reading frames in *E. coli* verified protein expression from the wild-type locus of an epitope-tagged *mgrB* [33]. The 47 amino acid MgrB peptide could potentially have a type I (secretion) or type II (lipidation) signal sequence [34],[35]. Alternatively, the N-terminal hydrophobic stretch may function as a transmembrane domain [36] (Figure 3A). To determine the localization of MgrB, we analyzed cell envelope and soluble protein fractions by Western blotting with rabbit antisera raised against a peptide from the C-terminus of the protein. We were able to detect a protein between 4 and 7 kilodaltons in the whole-cell lysate and envelope fraction of cells expressing *mgrB*, but not in the soluble protein fraction (Figure 3B). The envelope fraction of cells expressing MgrB showed no detectable contamination with cytoplasmic or periplasmic proteins as assessed by Western blots for the cytoplasmic proteins CFP and YFP and the periplasmic protein beta-lactamase (top two panels of Figure 3B).

To further confirm membrane association, we constructed a fusion of GFP to the N-terminus of MgrB. The fusion is similar to wild-type MgrB in its ability to complement a deletion, as indicated by repression of PhoQ/PhoP signaling (Figure S2). Western blots show no evidence of cleavage or degradation of the fusion protein (data not shown). The fact that GFP is not cleaved from MgrB by a signal peptidase suggests that nascent MgrB is not secreted into the periplasm or lipidated. Fluorescence microscopy of cells expressing GFP-MgrB revealed a halo-like fluorescence at the cell boundary, suggesting localization to the envelope, whereas cells expressing GFP alone showed uniform fluorescence indicative of cytoplasmic localization (Figure 3C). When GFP is targeted to the periplasm through the Sec pathway, either as a secreted protein or as part of a membrane protein, it does not fold properly and fails to fluoresce [37],[38]. The only reported pathway for producing fluorescent GFP in the periplasm is through the Tat secretion system [39],[40]. However MgrB is not predicted to have a signal sequence that would target it for secretion through this pathway [41]. Taken together, the above results indicate that MgrB is associated with the inner-membrane with its N-terminus in the cytoplasm. The size and hydrophobicity profile of the peptide further suggest that it spans the membrane with a single transmembrane domain.

### MgrB interaction with PhoQ

**Figure 4:**
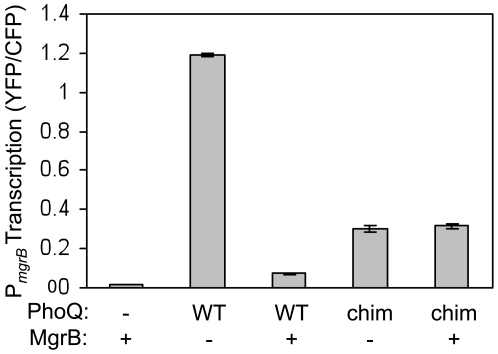
Alteration of the PhoQ periplasmic domain blocks the repressive effect of MgrB. Fluorescence of an *mgrB* transcriptional reporter, AML21 (*mgrB* ^−^ *phoQ* ^−^), which was transformed with a control plasmid (pEB52) or a plasmid containing *mgrB* (pAL8) and with a compatible plasmid expressing wild-type PhoQ (pLPQ2), a chimera in which the periplasmic domain of *E coli* PhoQ was replaced with the corresponding domain from *P. aeruginosa* PhoQ (pLPQ*2), or a control plasmid (pGB2). Fluorescence was measured from cells grown at 30°C in minimal glucose medium with 100 µM MgSO_4_ as described in Materials and Methods. Cells were grown at 30°C for consistency with bacterial two-hybrid experiments—Figure 5 and Figure S3. Similar results were observed for cells grown at 37°C (data not shown). The error bars represent the range of means of two independent cultures. (WT: wild-type, chim: chimera).

Given the localization of MgrB, we hypothesized that it exerts its effects through PhoQ. We therefore tested the action of MgrB on a chimera in which the periplasmic domain of PhoQ from *E. coli* was replaced with the highly divergent periplasmic domain of PhoQ from *Pseudomonas aeruginosa* [42], a bacterium that does not appear to possess an *mgrB* ortholog. We measured fluorescence of a *phoQ^−^ mgrB^−^* reporter strain for PhoP-dependent transcription that was transformed with a plasmid expressing either *E. coli* PhoQ or the PhoQ chimera (PhoQ_chim_), or a control plasmid. The strain also contained either a compatible plasmid expressing *mgrB* or a compatible control plasmid (Figure 4). We note that the fluorescence levels of the MgrB^−^ PhoQ^+^_chim_ strain were lower than the corresponding levels for the MgrB^−^ PhoQ^+^ strain, but they were significantly higher than the fluorescence levels of the PhoQ^−^ strain. As with the complementation experiments above, introduction of the MgrB expression plasmid resulted in decreased fluorescence of PhoQ^+^ cells. In contrast, no change in fluorescence levels was observed when the plasmid was introduced into the PhoQ^+^_chim_ strain. That MgrB does not decrease PhoP-regulated transcription when the periplasmic sensor domain of PhoQ is modified suggests MgrB acts at or upstream of PhoQ in the signaling pathway.

**Figure 5:**
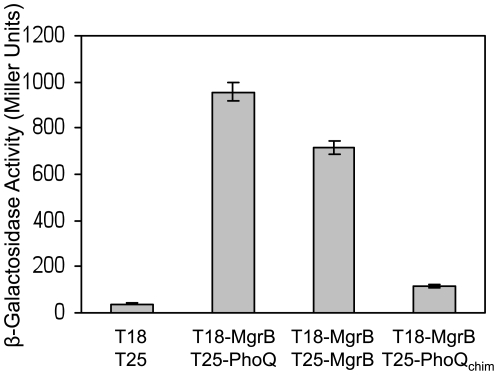
MgrB interacts with PhoQ and with itself. MgrB interacts with *E. coli* PhoQ and with itself but not with a PhoQ chimera containing a modified periplasmic domain. Cells expressed protein fusions to the T18 and T25 subunits of *B. pertussis* adenylyl cyclase. Reconstitution of adenylyl cyclase activity was inferred from beta-galactosidase activity. The *cyaA* ^−^ strain BTH101 contained plasmids expressing subunits alone (pKT25 or pUT18C) or subunit fusions to the N-terminus of MgrB (pAL25 and pAL33), *E. coli* PhoQ (pAL27), or a PhoQ chimera (PhoQ_chim_—pAL36). For each strain, the mean and standard deviation for three independent measurements are shown.

To look for evidence of an interaction between MgrB and PhoQ in *E. coli*, we used a bacterial two-hybrid assay based on split adenylyl cyclase [43]. In this system, adenylyl cyclase activity is reconstituted when the T18 and T25 fragments of *Bordetella pertussis* CyaA are brought into close proximity. The resulting increase in cAMP levels is detected through expression of beta-galactosidase from the *lac* promoter. We fused the T18 and T25 fragments to the N-termini of MgrB and PhoQ, respectively. Based on the known topology of PhoQ, and the topology of MgrB (discussed above), both CyaA fragments should be in the cytoplasm. A strain expressing T18-MgrB and T25-PhoQ showed a significantly higher level of beta-galactosidase activity when compared with strains expressing the T18 and T25 fragments alone (Figure 5) or expressing the fusions to either MgrB or PhoQ individually (data not shown). These results suggest there is a physical interaction between MgrB and PhoQ. Furthermore, a strain expressing T18-MgrB and the T25- fragment fused to the N-terminus of PhoQ_chim_ showed a minimal increase in beta-galactosidase activity relative to the controls. This is unlikely to be due to a defect in PhoQ_chim_ expression because we were able to detect PhoQ_chim_–PhoQ_chim_ interactions at levels comparable to those for (wild-type) PhoQ-PhoQ interactions, consistent with previous reports that both PhoQ and PhoQ_chim_ form functional complexes [42] (Figure S3). Taken together, these results suggest that the periplasmic domain of *E. coli* PhoQ is important for the interaction with MgrB. Interestingly, co-expression of T18-MgrB and a fusion of T25 to the N-terminus of MgrB also showed significant beta-galactosidase activity relative to the controls (Figure 5), suggesting that MgrB may form dimers or higher order complexes.

### MgrB homologs in other species

**Figure 6:**
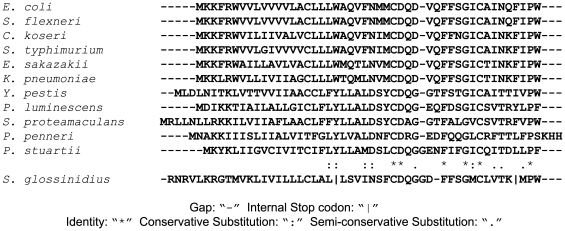
MgrB homologs identified from genome sequences of various genera of Enterobacteriaceae. The first 11 sequences were aligned using ClustalW [54]. Genome sequences were from *Escherichia coli* MG1655, *Shigella flexneri* 2457T, *Citrobacter koseri* ATCC BAA-895, *Salmonella typhimurium* LT2, *Enterobacter sakazakii* ATCC BAA-894, *Klebsiella pneumoniae* 342, *Yersinia pestis* KIM, *Photorhabdus luminescens* subsp. laumondii TTO1, *Serratia proteamaculans* 568, *Providencia stuartii* ATCC 25827, *Sodalis glossinidius* str. ‘morsitans’, and *Proteus penneri* ATCC 35198.

The *mgrB* gene, also known as *yobG*, has been identified in *Salmonella enterica*, where it has been shown to be activated by PhoP in microarray and transcriptional reporter experiments [11],[44]. Like many other small open reading frames, *mgrB* is frequently missed in genome annotations. However we have been able to identify putative *mgrB* homologs in numerous species among several genera of Enterobacteriaceae. Specific examples and their alignments are shown in Figure 6. All of the genomes for which we could identify a candidate *mgrB* also contained *phoP* and *phoQ* orthologs. However, the converse does not appear to be the case: we were unable to find an *mgrB* homolog in *Erwinia* or *Pseudomonas* species, though members of both of these genera possess *phoP* and *phoQ*. In addition, although the endosymbiont *Sodalis glossinidus* has *phoP* and *phoQ* genes, its *mgrB* is unlikely to be functional due to the presence of two internal stop codons (Figure 6).

**Figure 7:**
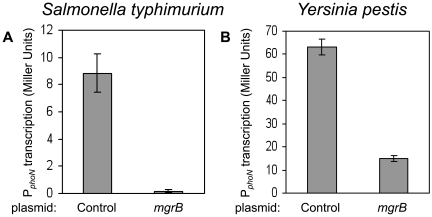
Inhibition of PhoP-regulated transcription by MgrB in *Salmonella typhimurium* and *Yersinia pestis*. (A) *Salmonella enterica* serovar Typhimurium strain 14028s carrying the P*_phoN_*-*lacZ* reporter plasmid pNL2 and either a plasmid expressing *Salmonella mgrB* (pAL43) or a control plasmid (pEB52) were grown in LB with antibiotics and 1 mM IPTG. The amino acid sequence of MgrB from strain 14028s (data not shown) is identical to the sequence from strain LT2 (shown in Figure 6). (B) *Y. pestis* KIM6 carrying pNL2 and either a plasmid expressing the *Yersinia pestis mgrB* (pAL42) or a control plasmid (pEB52) were grown in BHI with antibiotics and 1mM IPTG. For each strain, the mean and standard deviation for at least three independent cultures are shown.

To test whether the action of MgrB on PhoQ/PhoP signaling is conserved among several genera, we over-expressed the native MgrB from *Salmonella enterica* serovar typhimurium and *Yesinia pestis* in their respective host strains and measured transcription of the promoter for *phoN*, a PhoP-regulated gene from *Salmonella*. For both *Y. pestis* and *S. typhimurium*, MgrB over-expression resulted in a significant repression of reporter activity (Figure 7). We also verified that the *mgrB* genes from both of these organisms complement an *mgrB* deletion in *E. coli* (data not shown).

## Discussion

**Figure 8:**
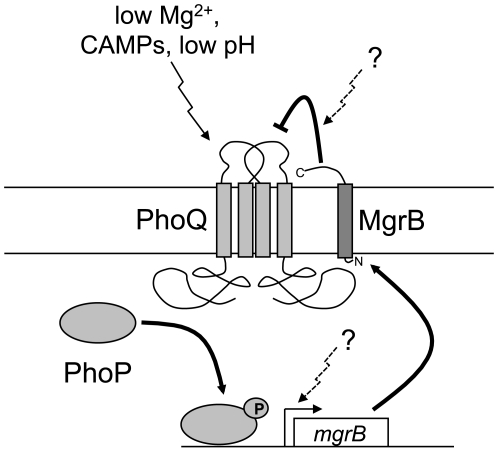
MgrB mediates negative feedback in the PhoQ/PhoP system. PhoQ stimulation by low extracellular magnesium or cationic antimicrobial peptides (CAMPs) leads to increased levels of phosphorylated PhoP, which in turn results in increased transcription of *mgrB*. MgrB inserts in the inner membrane, with its N-terminus in the cytoplasm and C-terminus in the periplasm, and represses PhoQ, resulting in decreased PhoP phosphorylation. The question marks illustrate potential points of control where additional signals could modulate PhoQ/PhoP signaling by regulating MgrB activity or expression.

Based on the results presented here, we propose that the peptide MgrB spans the inner-membrane and represses PhoP phosphorylation by inhibiting PhoQ kinase activity, stimulating phosphatase activity, or both (Figure 8). Our results further suggest that an interaction between MgrB and the periplasmic domain of PhoQ plays a critical role. Since *mgrB* transcription is activated by phosphorylated PhoP, MgrB is part of a negative feedback loop in the PhoQ/PhoP signaling circuit. It is particularly striking that deletion of *mgrB* results in a strong increase in PhoP-regulated transcription even for growth in 10 mM Mg^2+^–a condition that strongly represses PhoQ/PhoP signaling in wild-type cells. The four PhoP-regulated promoters that we tested (promoters for *mgrB*, *phoPQ*, *mgtA*, and *hemL*) show at least some level of magnesium responsiveness in an *mgrB* ^−^ strain (Figure 1B–1D and data not shown). The significant decrease in magnesium sensitivity of *mgtA* transcription in the absence of MgrB (Figure 1D) most likely indicates that *mgtA* has reached near-maximal levels of PhoP-activated transcription for the *mgrB* ^−^ strain growing in 10 mM Mg^2+^. Indeed, previous studies suggest that the *E. coli mgtA* promoter saturates at lower levels of PhoQ/PhoP stimulation when compared to the other promoters considered here [31]. We have also found that PhoP-regulated transcription remains responsive to the antimicrobial peptide LL37 and to acidic pH in *mgrB* ^−^ strains (Figure S4 and Figure S5, respectively). We note, however, that *mgrB* deletion affects the fold-change in reporter gene expression for all three stimuli (Mg^2+^, pH, and LL37). Thus, MgrB modulates the magnitude and sensitivity of PhoQ/PhoP signaling but is not strictly required for magnesium, pH, and antimicrobial peptide responsiveness.

Negative feedback has been shown to increase activation kinetics and minimize cell-to-cell variability in some regulatory circuits [25]–[27]. Such behavior can be readily understood for simple examples of feedback arising from negative autogenous control. However it is difficult to predict the effects of negative feedback in more complex circuits with additional regulators, which have the potential for time delays and additional sources of cell-to-cell variability. Indeed, deletion of MgrB has relatively little effect on the kinetics of fluorescent protein reporter accumulation (Figure S6) and on the cell-to-cell variability in fluorescent protein expression (Figure S7), despite its strong effect on the magnitude of PhoQ/PhoP signaling. We hypothesize that MgrB may provide a point of control for integrating additional input signals that modulate PhoQ/PhoP activity. For example, such signals could act by regulating the expression level or repressive action of MgrB (Figure 8).

We have shown that for bacteria from at least three different genera, the native MgrB homolog represses PhoQ/PhoP signaling, suggesting that this mode of negative regulation is broadly conserved. At least two other proteins have been reported to regulate PhoQ/PhoP activity, however the level of functional conservation appears to be more limited for these cases. YneN (B1500) is a small integral-membrane protein that stimulates PhoQ in *E. coli* [45]. This protein is a member of the EvgS/EvgA regulon and thus mediates cross-regulation between two signaling circuits. This mechanism for PhoQ regulation does not appear to be widely conserved; we have been unable to identify YneN orthologs in sequenced genomes other than those of various *E. coli* and *Shigella* isolates. Another protein, SlyB, has been shown to mediate some negative repression of the PhoQ/PhoP system in *Salmonella typhimurium*. Deletion of *slyB* resulted in a roughly 1.5-fold increase in transcription of multiple PhoP-regulated genes in this bacterium [44]. However, we did not detect any difference in transcription of PhoP-regulated genes when we compared wild-type and *slyB* ^−^ strains of *E. coli* (Figure 1B). This may indicate another example of divergence in the PhoQ/PhoP regulatory circuit, even for these closely related species [29],[46].

MgrB-mediated regulation of PhoQ is part of an emerging theme of modulation of membrane proteins by small hydrophobic peptides [47]. It is a striking example of a small, easily overlooked open reading frame that plays a critical role in regulating a signal transduction pathway. Sequence alignments reveal several conserved residues, which may be critical for MgrB function (Figure 6). Further genetic and biochemical work will be required to understand the mechanism by which MgrB represses PhoQ, and to determine whether MgrB has additional roles as part of this important two-component signaling system.

## Materials and Methods

See Supporting Information for tables of strains (Table S1), plasmids (Table S2), and PCR primers (Table S3). A description of plasmid construction is given in Text S1.

### Bacterial strains and growth conditions

Deletions of PhoP-regulated genes in *E. coli* were transduced from strains in the Keio Collection [48] by P1_vir_ transduction. The deletions were confirmed by PCR using primers that flank the gene. When necessary, kanamycin resistance markers were removed with FLP recombinase by transforming with pCP20 [49] and subsequent curing of the plasmid.

*E. coli* strains were grown at 37°C, unless otherwise indicated, in Luria-Bertani (LB) medium (Difco - BD, Franklin Lakes, NJ) or minimal A medium [50] supplemented with 0.2% glucose, 0.1% casamino acids (Difco) and with the indicated concentration of MgSO_4_. *Salmonella* strains were grown in LB at 37°C. *Yersinia pestis* strains were grown at 26°C in brain-heart infusion (BHI) broth (Difco). The *lac* and *trc* promoters were induced with isopropyl β-D-1-thiogalactopyranoside (IPTG) at a final concentration of 1 mM when indicated. When IPTG was not mentioned in the description of the culture conditions, the basal transcription from the *trc* promoter was used to drive expression.

### Fluorescence images of agar plates

Strains were streaked onto LB Agar plates (Difco) and incubated overnight at 37°C. Images of YFP and CFP fluorescence were acquired with a home-built fluorescence illuminator as previously described [51].

### Fluorescence microscopy and single-cell measurements

Single-cell measurements were performed essentially as described previously [31]. Briefly, overnight cultures, grown in minimal A medium with 1 mM MgSO_4_, and 50 µg/mL ampicillin with or without 50 µg/mL spectinomycin to maintain plasmids when necessary, were diluted back 1∶1000 in pre-warmed minimal medium containing 100 µM, 1 mM, or 10 mM MgSO_4_ and grown to an OD_600_ between 0.2 and 0.3. Cultures were cooled quickly with an ice water slurry and streptomycin was added to a final concentration of 250 µg/mL to inhibit protein synthesis. Images were acquired and analyzed as previously described [31],[32].

### Envelope fractionation

Envelopes were prepared with a protocol modified from [52]. An overnight culture in LB medium with 50 µg/mL ampicillin was diluted 1∶1000 into the same medium supplemented with 1mM IPTG and shaken at 37 degrees for 5.5 hours at 250 rpm. The culture was split in half, chilled on ice, and then spun at 3,300 g for 10 minutes at 4°C. One pellet was saved at −20°C for the total lysate. The second pellet was resuspended in a 10 mL of 30mM Tris pH 7.8 and pelleted again. The pellet was then resuspended by vortexing in 200 µL of a cold 20% sucrose/30mM Tris solution. 20 µL of freshly prepared 10 mg/mL lysozyme in 0.1 M EDTA pH 7.0 was added and the solution was mixed by inverting at 4°C for 20 minutes. 3 mL of 3 mM EDTA pH 7.5 was then added, the solution was sonicated on ice, and then centrifuged at 47,000 g at 4°C for 75 minutes. The supernatant, which is the soluble fraction, was removed and saved. The pellet, which is the envelope fraction, was resuspended in 2 mM Tris pH 8.0. To bring the volume of supernatant down to the same concentration of cell equivalents as the envelope fraction, the supernatant was concentrated with a Speed Vac (Thermo Fisher, Waltham, MA).

### Western blot analysis

Samples were boiled in Tris-Tricine loading buffer with 0.2 M dithiothreitol for 5–7 minutes and loaded on 16.5% Tris tricine gels (ReadyGel - BioRad, Hercules, CA). Proteins were transferred to Immobilon-P PVDF (Millipore, Billerica, MA) followed by western blot analysis. A rabbit polyclonal MgrB antiserum was generated by using a KLH-conjugated synthetic peptide corresponding to residues 27–40 of the predicted MgrB protein sequence (GenScript, Piscataway, NJ). The peptide was synthesized with a C-to-S substitution for the first cysteine residue. Beta-lactamase and CFP/YFP were detected with rabbit polyclonal anti-beta-lactamase (Millipore,) and anti-GFP (A.v. Peptide Antibody – Clontech, Mountain View, CA) antibodies. A horseradish peroxidase-conjugated anti-rabbit antiserum (GE Healthcare, Piscataway, NJ) was used as the secondary antibody.

### Bacterial two-hybrid assays

Cultures of BTH101 bearing combinations of pKT25- and pUT18-derived plasmids were grown for 9 hours in LB supplemented with 100 µg/mL ampicillin and 50 µg/mL kanamycin. The cultures were then diluted 1∶1000 into medium supplemented with 1 mM IPTG and allowed to grow at 30°C for 14 hours. Cultures were grown at 30°C in order to increase complementation efficiency [53]. Cells were cooled quickly in an ice slurry and kept on ice for 30 minutes. Beta-galactosidase assays were performed as described in [50] using chloroform and SDS for permeabilization.

### Beta-galactosidase measurements for *Salmonella* and *Yersinia*

Cultures of *Salmonella* were grown overnight in LB with 50 µg/mL ampicillin and 50 µg/mL spectinomycin. The next day, they were diluted 1∶1000 into the same medium and grown to OD_600_ 0.1–0.3. Cells were permeabilized with chloroform and SDS and assayed as in [50]. Cultures of *Yersina* were grown and assayed in the same fashion except the culture medium was BHI with 200 µg/mL ampicillin and 50 µg/mL spectinomycin and the overnight culture was diluted 1∶50.

## Supporting Information

Figure S1 Of seven PhoP-regulated genes, only deletion of *mgrB* leads to increased reporter expression compared with wild-type. Each of seven PhoP-regulated genes were deleted individually in the *mgrB* transcriptional reporter strain TIM92 and examined for fold-change in YFP/CFP fluorescence relative to the wild-type strain TIM92 (WT). The *phoQ* deletion (Δ*phoQ*) is shown for reference. Cultures were grown in minimal A medium with 100 µM MgSO_4_ (A) or 10 mM MgSO_4_ (B) and analyzed by fluorescence microscopy as described in Materials and Methods. Strains are, from left to right, TIM92, TIM99, AML6, AML8, AML10, AML12, AML14, AML16.
(0.20 MB PDF)

Figure S2 GFP-MgrB complements an mgrB deletion. P*_mgtA_*-*lacZ* expression is shown for wild-type and *mgrB* ^−^ strains containing a control plasmid (left and middle columns, respectively) and an *mgrB* ^−^ strain expressing GFP-MgrB (right column). Cultures were grown overnight in LB with 50 µg/mL ampicillin, diluted back 1∶1000 into pre-warmed medium, and grown at 37°C for 4 hours. Beta-galactosidase assays were performed as described in Materials and Methods. For each strain, the means and standard deviations for three independent measurements are shown. Strains are, from left to right, pEB52/TIM199, pAL39/AML67, and pAL38/AML67.
(0.20 MB PDF)

Figure S3 PhoQ-PhoQ and PhoQchim-PhoQchim interactions can be detected by a bacterial two-hybrid assay. The *cyaA* ^−^ *phoQ* ^−^ strain AML69 contained combinations of plasmids expressing adenylyl cyclase subunits T18 (pUT18) and T25 (pKT25), fusions of the T25 subunit to the N-terminus of PhoQ (pAL27) or PhoQ_chim_ (pAL36), and fusions of the T18 subunit to the C-terminus of PhoQ (pAL41) or PhoQ_chim_ (pAL46) as indicated. Cells were grown and beta-galactosidase assays were performed as described in Materials and Methods. For each strain, the means and standard deviations for three independent measurements are shown.
(0.20 MB PDF)

Figure S4 The antimicrobial peptide LL-37 affects PhoP-regulated transcription in the absence of MgrB. Transcriptional reporters for the *phoPphoQ* operon were grown in the presence or absence of LL37. Cells were grown in minimal A medium with 1 mM MgSO_4_, 0.2% glucose, and 0.1% casamino acids overnight at 37°C. Overnight cultures were then diluted 1∶1000 into tubes with pre-warmed medium containing 100 µM MgSO_4_. Cells were grown for 3.5 hours before cultures were split into two tubes and one received LL37 peptide (which was a gift from P. Janmey, University of Pennsylvania School of Medicine) to a final concentration of 2.5 µg/mL and the other received diluent alone. Cultures were allowed to grow one additional hour and then were treated as described in Materials and Methods for fluorescence microscopy and single-cell measurements. Error bars represent the range of means for two independent experiments. Strains are from left to right TIM148, TIM229, and AML22.
(0.27 MB PDF)

Figure S5 Low pH affects PhoP-regulated transcription in the absence of MgrB. Transcriptional reporters of *mgrB* expression grown overnight in MES-buffered N-minimal medium pH 7.5 (5 mM KCl, 7.5 mM (NH_4_)_2_SO_4_, 0.5 mM K_2_SO_4_, 1 mM KH_2_PO_4_, 100 mM MES) supplemented with 10 mM MgCl_2_, 0.2% glucose, and 0.1% casamino acids were diluted 1∶1000 into medium of pH 5.5 or pH 7.5 and allowed to grow to mid-log. Cells were then treated as described in Materials and Methods for fluorescence microscopy and single-cell measurements. Error bars represent the range of means for two independent experiments. Strains are from left to right TIM92, TIM100, and AML20.
(0.20 MB PDF)

Figure S6 Kinetics of reporter induction for *mgrB* ^+^ and *mgrB* ^−^ strains. Transcriptional reporters for the *phoPphoQ* operon were grown in repressing conditions (10 mM MgSO_4_) and then shifted to inducing (100 µM MgSO_4_) medium and allowed to grow. Reporter transcription in wild-type and *mgrB* ^−^ cells is shown as raw data (A) and as curves normalized by shifting and rescaling as indicated below (B). Cells were grown in minimal A medium with 10 mM MgSO_4_, 0.2% glucose, and 0.1% casamino acids overnight at 37°C. Overnight cultures were then diluted 1∶1000 into tubes with pre-warmed medium containing 10 mM MgSO_4_. 2 mL cultures were then grown for 4 hours before being spun down and resuspended in 2 mL medium with 100 µM MgSO_4_. Cultures were allowed to grow for 4 hours, with aliquots removed and treated with streptomycin and immediately placed in an ice water slurry at 2 minutes prior to induction and at the indicated times after induction. Cells remained on ice for 1 hour and then analyzed by fluorescence microscopy as described in Materials and Methods. For (B), fluorescence was normalized as follows: fl_normalized_(t) = [fl(t)-fl(t_initial_)]/[fl(t_final_)-fl(t_initial_)], where fl(t) denotes the fluorescence at time t minutes (labeled as P*_phoPQ_* transcription in the figure) and t_initial_ and t_final_ denote the first and last time points. Results from a single representative experiment are shown. Strains are TIM148 (diamonds) and AML53 (squares).
(0.40 MB PDF)

Figure S7 The distribution of single-cell YFP/CFP fluorescence for *mgrB^+^* and *mgrB^−^* cells. PmgrB fluorescent reporter strains were grown in 100 µM MgSO_4_ (A) or 10 mM MgSO_4_ (B) and analyzed by fluorescence microscopy as described in Materials and Methods. For each culture, values of cellular YFP/CFP were normalized by the mean of the distribution. For the 100 µM Mg^2+^ cultures, the mean (μ), and coefficient of variation (standard deviation/mean, c_v_) for YFP/CFP were μ = 0.84, c_v_ = 0.19 and μ = 2.75, c_v_ = 0.13 for *mgrB^+^* and *mgrB^−^* strains, respectively. For the 10 mM Mg2+ cultures, μ = 0.13, cv = 0.26 and μ = 1.3, cv = 0.19 for *mgrB_+_* and *mgrB^−^* strains, respectively. Each distribution represents a sample of at least 140 cells. Strains are TIM92 (white) and AML16 (black).
(0.26 MB PDF)

Table S1 Strains.
(0.12 MB DOC)

Table S2 Plasmids.
(0.08 MB DOC)

Table S3 Primers.
(0.04 MB DOC)

Text S1 Plasmid construction.
(0.04 MB DOC)
