# Supplementary material for: Feedback Inhibition in the PhoQ/PhoP Signaling System by a Membrane Peptide
Source: PLoS Genet. 2009 Dec 24;5(12):e1000788. doi: 10.1371/journal.pgen.1000788 (PMC2789325; doi:10.1371/journal.pgen.1000788)
Supplement: Figure S1 — Of seven PhoP-regulated genes, only deletion of mgrB leads to increased reporter expression compared with wild-type. Each of seven PhoP-regulated genes were deleted individually in the mgrB transcriptional reporter strain TIM92 and examined for fold-change in YFP/CFP fluorescence relative to the wild-type strain TIM92 (WT). The phoQ deletion (ΔphoQ) is shown for reference. Cultures were grown in minimal A medium with 100 µM MgSO4 (A) or 10 mM MgSO4 (B) and analyzed by fluorescence microscopy as described in Materials and Methods. Strains are, from left to right, TIM92, TIM99, AML6, AML8, AML10, AML12, AML14, AML16. (0.20 MB PDF) [file pgen.1000788.s001.pdf]

A

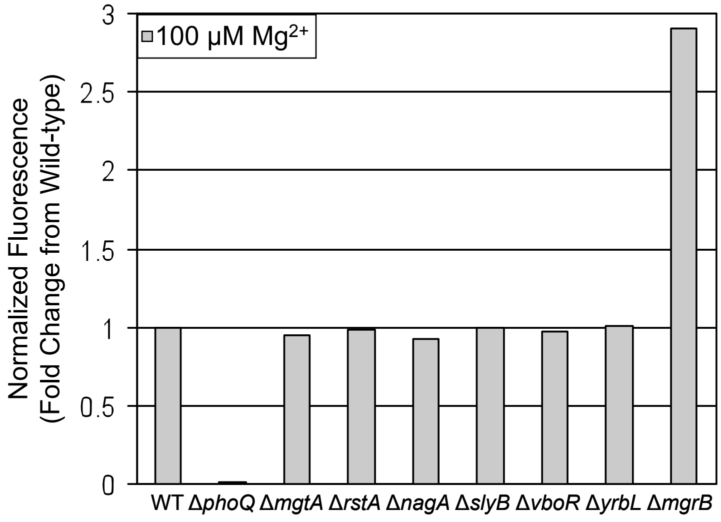

B

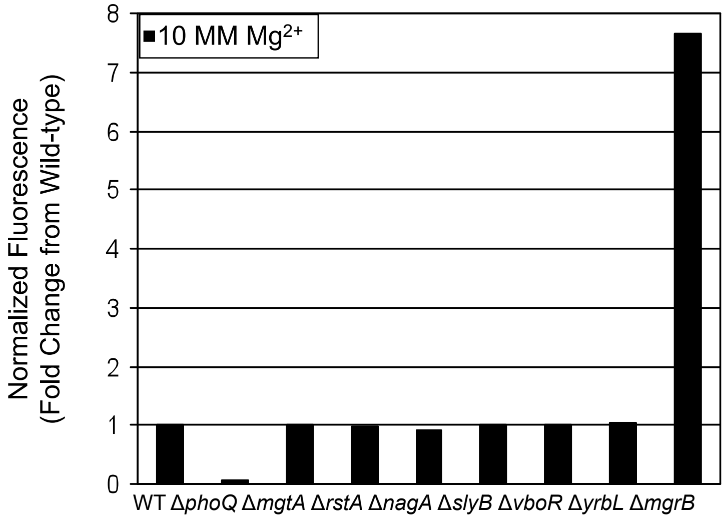

Figure S1. Of seven PhoP-regulated genes, only deletion of *mgrB* leads to increased reporter expression compared with Wild-type.

Each of seven PhoP-regulated genes were deleted individually in the *mgrB* transcriptional reporter strain TIM92 and examined for fold-change in YFP/CFP fluorescence relative to the wild-type strain TIM92 (WT). The *phoQ* deletion (Δ*phoQ*) is shown for reference. Cultures were grown in minimal A medium with 100 μM $MgSO_4$ (A) or 10 mM $MgSO_4$ (B) and analyzed by fluorescence microscopy as described in Materials and Methods. Strains are, from left to right, TIM92, TIM99, AML6, AML8, AML10, AML12, AML14, AML16.
