# Supplementary material for: Feedback Inhibition in the PhoQ/PhoP Signaling System by a Membrane Peptide
Source: PLoS Genet. 2009 Dec 24;5(12):e1000788. doi: 10.1371/journal.pgen.1000788 (PMC2789325; doi:10.1371/journal.pgen.1000788)
Supplement: Figure S2 — GFP-MgrB complements an mgrB deletion. PmgtA-lacZ expression is shown for wild-type and mgrB − strains containing a control plasmid (left and middle columns, respectively) and an mgrB − strain expressing GFP-MgrB (right column). Cultures were grown overnight in LB with 50 µg/mL ampicillin, diluted back 1∶1000 into pre-warmed medium, and grown at 37°C for 4 hours. Beta-galactosidase assays were performed as described in Materials and Methods. For each strain, the means and standard deviations for three independent measurements are shown. Strains are, from left to right, pEB52/TIM199, pAL39/AML67, and pAL38/AML67. (0.20 MB PDF) [file pgen.1000788.s002.pdf]

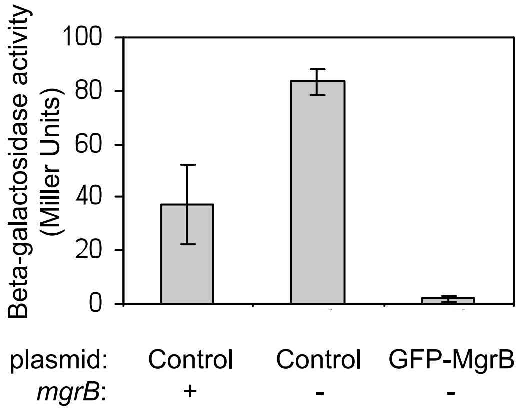

Figure S2. GFP-MgrB complements an *mgrB* deletion.
P_{mgtA}-*lacZ* expression is shown for wild-type and *mgrB*$^-$ strains containing a control plasmid (left and middle columns, respectively) and an *mgrB*$^-$ strain expressing GFP-MgrB (right column). Cultures were grown overnight in LB with 50 µg/mL ampicillin, diluted back 1:1000 into pre-warmed medium, and grown at 37°C for 4 hours. Beta-galactosidase assays were performed as described in Materials and Methods. For each strain, the means and standard deviations for three independent measurements are shown. Strains are, from left to right, pEB52/TIM199, pAL39/AML67, and pAL38/AML67.
