# Supplementary material for: Feedback Inhibition in the PhoQ/PhoP Signaling System by a Membrane Peptide
Source: PLoS Genet. 2009 Dec 24;5(12):e1000788. doi: 10.1371/journal.pgen.1000788 (PMC2789325; doi:10.1371/journal.pgen.1000788)
Supplement: Figure S3 — PhoQ-PhoQ and PhoQchim-PhoQchim interactions can be detected by a bacterial two-hybrid assay. The cyaA − phoQ − strain AML69 contained combinations of plasmids expressing adenylyl cyclase subunits T18 (pUT18) and T25 (pKT25), fusions of the T25 subunit to the N-terminus of PhoQ (pAL27) or PhoQchim (pAL36), and fusions of the T18 subunit to the C-terminus of PhoQ (pAL41) or PhoQchim (pAL46) as indicated. Cells were grown and beta-galactosidase assays were performed as described in Materials and Methods. For each strain, the means and standard deviations for three independent measurements are shown. (0.20 MB PDF) [file pgen.1000788.s003.pdf]

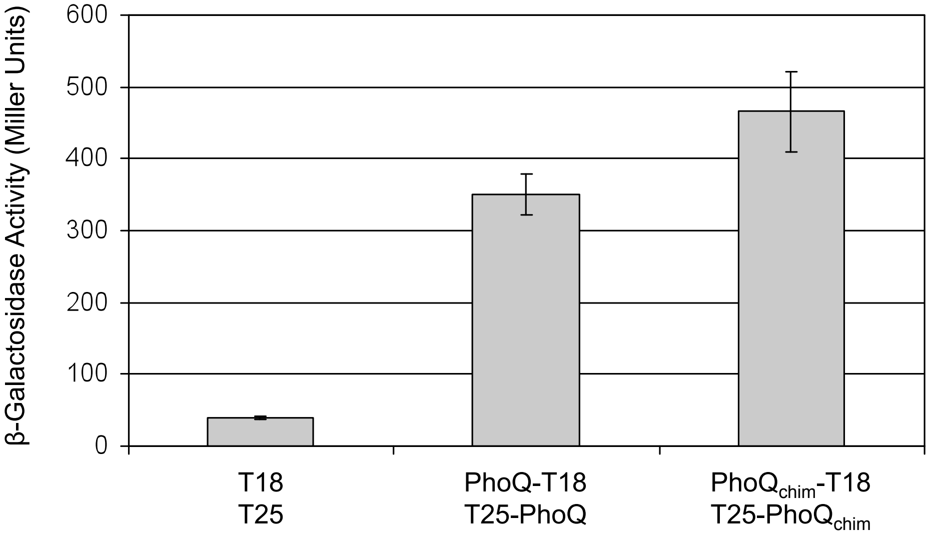

Figure S3. PhoQ-PhoQ and PhoQ$_{chim}$-PhoQ$_{chim}$ interactions can be detected by a bacterial two-hybrid assay. The *cyaA*$^{-}$ *phoQ*$^{-}$ strain AML69 contained combinations of plasmids expressing adenylyl cyclase subunits T18 (pUT18) and T25 (pKT25), fusions of the T25 subunit to the N-terminus of PhoQ (pAL27) or PhoQ$_{chim}$ (pAL36), and fusions of the T18 subunit to the C-terminus of PhoQ (pAL41) or PhoQ$_{chim}$ (pAL46) as indicated. Cells were grown and beta-galactosidase assays were performed as described in Materials and Methods. For each strain, the means and standard deviations for three independent measurements are shown.
