# Supplementary material for: Feedback Inhibition in the PhoQ/PhoP Signaling System by a Membrane Peptide
Source: PLoS Genet. 2009 Dec 24;5(12):e1000788. doi: 10.1371/journal.pgen.1000788 (PMC2789325; doi:10.1371/journal.pgen.1000788)
Supplement: Figure S4 — The antimicrobial peptide LL-37 affects PhoP-regulated transcription in the absence of MgrB. Transcriptional reporters for the phoPphoQ operon were grown in the presence or absence of LL37. Cells were grown in minimal A medium with 1 mM MgSO4, 0.2% glucose, and 0.1% casamino acids overnight at 37°C. Overnight cultures were then diluted 1∶1000 into tubes with pre-warmed medium containing 100 µM MgSO4. Cells were grown for 3.5 hours before cultures were split into two tubes and one received LL37 peptide (which was a gift from P. Janmey, University of Pennsylvania School of Medicine) to a final concentration of 2.5 µg/mL and the other received diluent alone. Cultures were allowed to grow one additional hour and then were treated as described in Materials and Methods for fluorescence microscopy and single-cell measurements. Error bars represent the range of means for two independent experiments. Strains are from left to right TIM148, TIM229, and AML22. (0.27 MB PDF) [file pgen.1000788.s004.pdf]

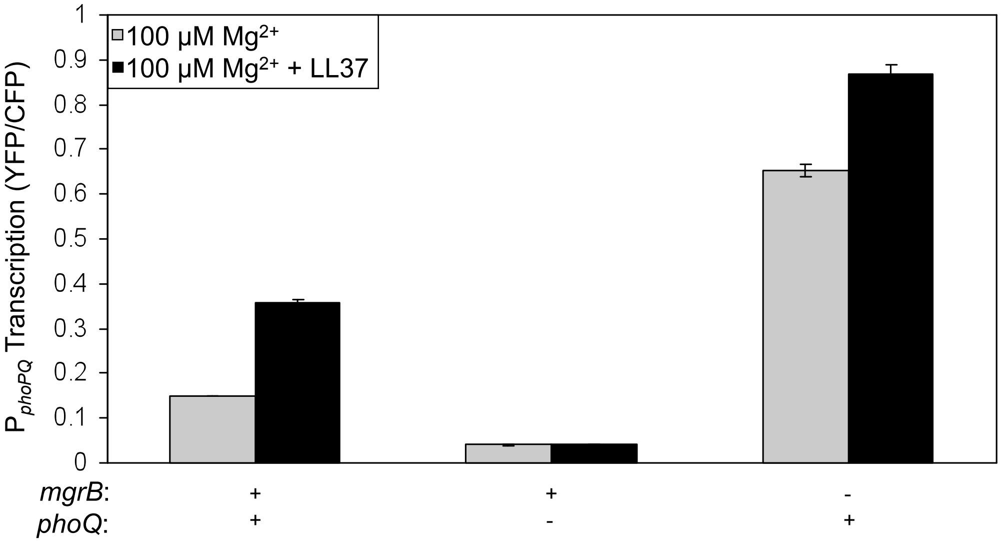

Figure S4. The antimicrobial peptide LL-37 affects PhoP-regulated transcription in the absence of MgrB. Transcriptional reporters for the *phoPphoQ* operon were grown in the presence or absence of LL37. Cells were grown in minimal A medium with 1 mM $MgSO_4$, 0.2% glucose, and 0.1% casamino acids overnight at 37°C. Overnight cultures were then diluted 1:1000 into tubes with pre-warmed medium containing 100 µM $MgSO_4$. Cells were grown for 3.5 hours before cultures were split into two tubes and one received LL37 peptide (which was a gift from P. Janmey, University of Pennsylvania School of Medicine) to a final concentration of 2.5 µg/mL and the other received diluent alone. Cultures were allowed to grow one additional hour and then were treated as described in Materials and Methods for fluorescence microscopy and single-cell measurements. Error bars represent the range of means for two independent experiments. Strains are from left to right TIM148, TIM229, and AML22.
