# Supplementary material for: Feedback Inhibition in the PhoQ/PhoP Signaling System by a Membrane Peptide
Source: PLoS Genet. 2009 Dec 24;5(12):e1000788. doi: 10.1371/journal.pgen.1000788 (PMC2789325; doi:10.1371/journal.pgen.1000788)
Supplement: Figure S5 — Low pH affects PhoP-regulated transcription in the absence of MgrB. Transcriptional reporters of mgrB expression grown overnight in MES-buffered N-minimal medium pH 7.5 (5 mM KCl, 7.5 mM (NH4)2SO4, 0.5 mM K2SO4, 1 mM KH2PO4, 100 mM MES) supplemented with 10 mM MgCl2, 0.2% glucose, and 0.1% casamino acids were diluted 1∶1000 into medium of pH 5.5 or pH 7.5 and allowed to grow to mid-log. Cells were then treated as described in Materials and Methods for fluorescence microscopy and single-cell measurements. Error bars represent the range of means for two independent experiments. Strains are from left to right TIM92, TIM100, and AML20. (0.20 MB PDF) [file pgen.1000788.s005.pdf]

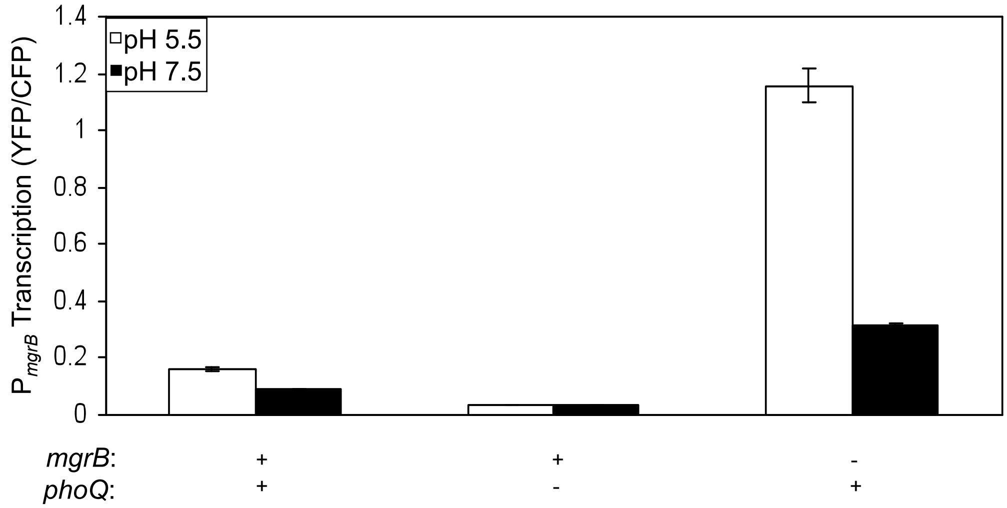

Figure S5. Low pH affects PhoP-regulated transcription in the absence of MgrB. Transcriptional reporters of *mgrB* expression grown overnight in MES-buffered N-minimal medium pH 7.5 (5 mM KCl, 7.5 mM $(NH_4)_2SO_4$, 0.5 mM K_2SO_4, 1 mM KH_2PO_4, 100 mM MES) supplemented with 10 mM $MgCl_2$, 0.2% glucose, and 0.1% casamino acids were diluted 1:1000 into medium of pH 5.5 or pH 7.5 and allowed to grow to mid-log. Cells were then treated as described in Materials and Methods for fluorescence microscopy and single-cell measurements. Error bars represent the range of means for two independent experiments. Strains are from left to right TIM92, TIM100, and AML20.
