# Supplementary material for: Feedback Inhibition in the PhoQ/PhoP Signaling System by a Membrane Peptide
Source: PLoS Genet. 2009 Dec 24;5(12):e1000788. doi: 10.1371/journal.pgen.1000788 (PMC2789325; doi:10.1371/journal.pgen.1000788)
Supplement: Figure S6 — Kinetics of reporter induction for mgrB + and mgrB − strains. Transcriptional reporters for the phoPphoQ operon were grown in repressing conditions (10 mM MgSO4) and then shifted to inducing (100 µM MgSO4) medium and allowed to grow. Reporter transcription in wild-type and mgrB − cells is shown as raw data (A) and as curves normalized by shifting and rescaling as indicated below (B). Cells were grown in minimal A medium with 10 mM MgSO4, 0.2% glucose, and 0.1% casamino acids overnight at 37°C. Overnight cultures were then diluted 1∶1000 into tubes with pre-warmed medium containing 10 mM MgSO4. 2 mL cultures were then grown for 4 hours before being spun down and resuspended in 2 mL medium with 100 µM MgSO4. Cultures were allowed to grow for 4 hours, with aliquots removed and treated with streptomycin and immediately placed in an ice water slurry at 2 minutes prior to induction and at the indicated times after induction. Cells remained on ice for 1 hour and then analyzed by fluorescence microscopy as described in Materials and Methods. For (B), fluorescence was normalized as follows: flnormalized(t) = [fl(t)-fl(tinitial)]/[fl(tfinal)-fl(tinitial)], where fl(t) denotes the fluorescence at time t minutes (labeled as PphoPQ transcription in the figure) and tinitial and tfinal denote the first and last time points. Results from a single representative experiment are shown. Strains are TIM148 (diamonds) and AML53 (squares). (0.40 MB PDF) [file pgen.1000788.s006.pdf]

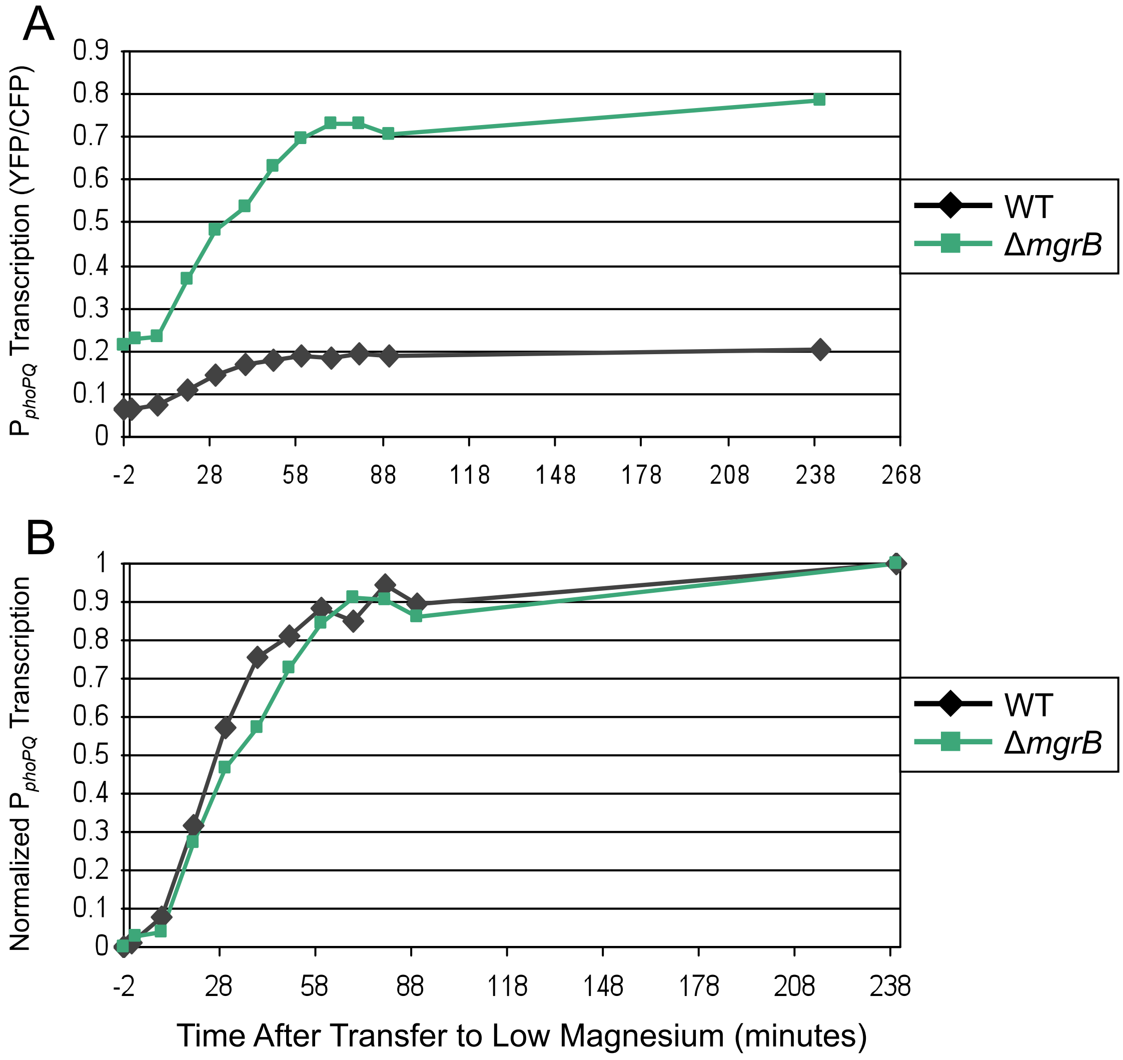

Figure S6. Kinetics of reporter induction for *mgrB*⁺ and *mgrB*⁻ strains. Transcriptional reporters for the *phoPphoQ* operon were grown in repressing conditions (10 mM $MgSO_4$) and then shifted to inducing (100 µM $MgSO_4$) medium and allowed to grow. Reporter transcription in wild-type and *mgrB*⁻ cells is shown as raw data (A) and as curves normalized by shifting and rescaling as indicated below (B). Cells were grown in minimal A medium with 10 mM $MgSO_4$, 0.2% glucose, and 0.1% casamino acids overnight at 37°C. Overnight cultures were then diluted 1:1000 into tubes with pre-warmed medium containing 10 mM $MgSO_4$. 2 mL cultures were then grown for 4 hours before being spun down and resuspended in 2 mL medium with 100 µM $MgSO_4$. Cultures were allowed to grow for 4 hours, with aliquots removed and treated with streptomycin and immediately placed in an ice water slurry at 2 minutes prior to induction and at the indicated times after induction. Cells remained on ice for 1 hour and then analyzed by fluorescence microscopy as described in Materials and Methods. For figure B, fluorescence was normalized as follows:

$fl_{normalized}(t)=[fl(t)-fl(t_{initial})]/[fl(t_{final})-fl(t_{initial})]$,

where fl(t) denotes the fluorescence at time t minutes (labeled as P_{phoPQ} transcription in the figure) and $t_{initial}$ and t_{final} denote the first and last time points. Results from a single representative experiment are shown. Strains are TIM148 (diamonds) and AML53 (squares).
