# Supplementary material for: Feedback Inhibition in the PhoQ/PhoP Signaling System by a Membrane Peptide
Source: PLoS Genet. 2009 Dec 24;5(12):e1000788. doi: 10.1371/journal.pgen.1000788 (PMC2789325; doi:10.1371/journal.pgen.1000788)
Supplement: Figure S7 — The distribution of single-cell YFP/CFP fluorescence for mgrB+ and mgrB− cells. PmgrB fluorescent reporter strains were grown in 100 µM MgSO4 (A) or 10 mM MgSO4 (B) and analyzed by fluorescence microscopy as described in Materials and Methods. For each culture, values of cellular YFP/CFP were normalized by the mean of the distribution. For the 100 µM Mg2+ cultures, the mean (μ), and coefficient of variation (standard deviation/mean, cv) for YFP/CFP were μ = 0.84, cv = 0.19 and μ = 2.75, cv = 0.13 for mgrB+ and mgrB− strains, respectively. For the 10 mM Mg2+ cultures, μ = 0.13, cv = 0.26 and μ = 1.3, cv = 0.19 for mgrB+ and mgrB− strains, respectively. Each distribution represents a sample of at least 140 cells. Strains are TIM92 (white) and AML16 (black). (0.26 MB PDF) [file pgen.1000788.s007.pdf]

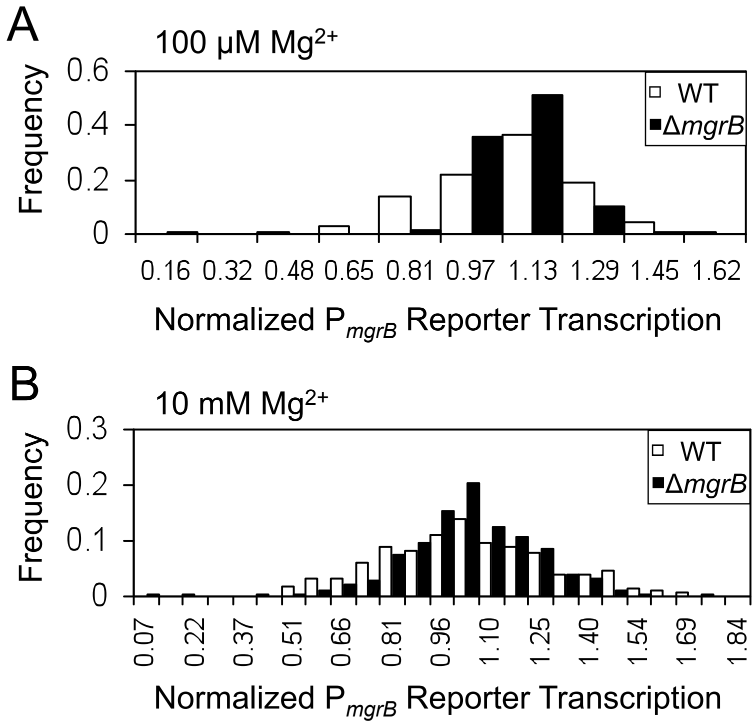

Figure S7. The distribution of single-cell YFP/CFP fluorescence for *mgrB*⁺ and *mgrB*⁻ cells.

P_{mgrB} fluorescent reporter strains were grown in 100 µM $MgSO_4$ (A) or 10 mM $MgSO_4$ (B) and analyzed by fluorescence microscopy as described in Materials and Methods. For each culture, values of cellular YFP/CFP were normalized by the mean of the distribution. For the 100 µM Mg^{2+} cultures, the mean (μ), and coefficient of variation (standard deviation/mean, c_v) for YFP/CFP were μ=0.84, c_v=0.19 and μ=2.75, c_v=0.13 for *mgrB*$^+$ and *mgrB*$^-$ strains, respectively. For the 10 mM Mg^{2+} cultures, μ=0.13, c_v=0.26 and μ=1.3, c_v=0.19 for *mgrB*$^+$ and *mgrB*$^-$ strains, respectively. Each distribution represents a sample of at least 140 cells. Strains are TIM92 (white) and AML16 (black).
