# Supplementary material for: Feedback Inhibition in the PhoQ/PhoP Signaling System by a Membrane Peptide
Source: PLoS Genet. 2009 Dec 24;5(12):e1000788. doi: 10.1371/journal.pgen.1000788 (PMC2789325; doi:10.1371/journal.pgen.1000788)
Supplement: Table S1 — Strains. (0.12 MB DOC) [file pgen.1000788.s008.doc]

**Table S1.** Strains

| Strains | Relevant Genotype | Source, reference, or construction* |
| --- | --- | --- |
| *Escherichia coli* strains | | |
| MG1655 | λ- rph-1 | *E. coli* genetic stock center no. 7740 |
| AML4 | TIM92 Δ(*mgtA*)::*kan* | P1(JW4201) X TIM92 |
| AML6 | TIM92 Δ(*rstA*)::*kan* | P1(JW1600) X TIM92 |
| AML8 | TIM92 Δ(*nagA*)::*kan* | P1(JW0663) X TIM92 |
| AML10 | TIM92 Δ(*slyB)::kan* | P1(JW1633) X TIM92 |
| AML11 | TIM100 Δ(*slyB*)::*kan* | P1(JW1633) X TIM100 |
| AML12 | TIM92 Δ(*vboR*)::*kan* | P1(JW0546) X TIM92 |
| AML14 | TIM92 Δ(*yrbL*)::*kan* | P1(JW3174) X TIM92 |
| AML16 | TIM92 Δ(*mgrB)::kan* | P1(JW1815) X TIM92 |
| AML17 | TIM100 Δ(*mgrB*)::*kan* | P1(JW1815) X TIM100 |
| AML20 | TIM92 Δ*mgrB*::FRT | AML16/pCP20 |
| AML21 | TIM100 Δ*mgrB*::FRT | AML17/pCP20 |
| AML22 | TIM148 (Δ*mgrB*)::*kan* | P1(JW1815) X TIM148 |
| AML23 | TIM229 (Δ*mgrB*)::*kan* | P1(JW1815) X TIM229 |
| AML24 | TIM91 (Δ*mgrB*)::*kan* | P1(JW1815) X TIM91 |
| AML25 | TIM99 (Δ*mgrB)::*kan | P1(JW1815) X TIM99 |
| AML54 | TIM229 Δ*mgrB*::FRT | AML23/pCP20 |
| AML67 | TIM199 (Δ*mgrB*)::*kan* | P1(JW1815) X TIM199 |
| AML68 | BTH101 Δ*phoQ::kan* | P1(JW1115) X BTH101 |
| AML69 | BTH101 Δ*phoQ*::FRT | This work |
| BTH101 | F-, cya-99, araD139, galE15, galK16, rpsL1 (Strr), hsdR2, mcrA1, mcrB1 | Euromedex, France. |
| BW25113 | *rrnB3* Δ*lacZ4787*  *hsdR514* Δ(*araBAD*)*567* Δ(*rhaBAD*)*568 rph-1* | [1] |
| JW0546 | BW25113 Δ*vboR*::*kan* | [2] |
| JW0663 | BW25113 Δ*nagA*::*kan* | [2] |
| JW1115 | BW25113 Δ*phoQ*::*kan* | [2] |
| JW1600 | BW25113 Δ*rstA*::*kan* | [2] |
| JW1633 | BW25113 Δ*slyB*::*kan* | [2] |
| JW1815 | BW25113 Δ*mgrB*::*kan* | [2] |
| JW3174 | BW25113 Δ*yrbL*::*kan* | [2] |
| JW4201 | BW25113 Δ*mgtA*::*kan* | [2] |
| TIM91 | MG1655 λatt::(P*mgtA-yfp*) HKatt::(P*tetA-cfp*) | [3] |
| TIM92 | MG1655 λatt::(P*mgrB*-*yfp*) HKatt::(P*tetA*-*cfp*) | [3] |
| TIM99 | MG1655 Δ*phoQ* λatt::(P*mgtA-yfp*) HKat::(P*tetA-cfp*) | [3] |
| TIM100 | MG1655 Δ*phoQ* λatt::(P*mgrB*-*yfp*) HKatt::(P*tetA*-*cfp*) | [3] |
| TIM148 | MG1655 λatt::(P*phoPQ*-*yfp cat*) HKatt­::(P*tetA*-*cfp*) | [3] |
| TIM199 | MG1655 Δ*lacZ* λatt::(P*mgtA*-*lacZ* *cat*) | Goulian lab stock |
| TIM229 | MG1655 Δ*phoQ* λatt::(P*phoPQ*-*yfp cat*) HKatt::­(P*tetA*-*cfp*) | [3] |
| *Salmonella enterica* subsp enterica serovar Typhimurium strains | | |
| 14028s  (table continues on next page) |  | D. Schifferli |
| *Yersinia pestis strains* | | |
| KIM6 | KIM5 pCD1- (LCR‑) *pgm* | [4], D. Schifferli |

* P1(AAA) X BBB denotes P1 transduction from strain AAA into strain BBB.

AAA/pCP20 denotes removal of kanamycin resistance from strain AAA by expression of FLP recombinase via pCP20 and subsequent curing of the plasmid.

1. Haldimann A, Wanner BL (2001) Conditional-Replication, Integration, Excision, and Retrieval Plasmid-Host Systems for Gene Structure-Function Studies of Bacteria. J Bacteriol 183: 6384-6393.

2. Baba T, Ara T, Hasegawa M, Takai Y, Okumura Y, et al. (2006) Construction of Escherichia coli K-12 in-frame, single-gene knockout mutants: the Keio collection. Mol Syst Biol 2: 2006.0008.

3. Miyashiro T, Goulian M (2007) Stimulus-dependent differential regulation in the Escherichia coli PhoQ PhoP system. Proc Natl Acad Sci U S A 104: 16305-16310.

4. Goguen JD, Yother J, Straley SC (1984) Genetic analysis of the low calcium response in Yersinia pestis mu d1(Ap lac) insertion mutants. J Bacteriol 160: 842-848.
