# Supplementary material for: Feedback Inhibition in the PhoQ/PhoP Signaling System by a Membrane Peptide
Source: PLoS Genet. 2009 Dec 24;5(12):e1000788. doi: 10.1371/journal.pgen.1000788 (PMC2789325; doi:10.1371/journal.pgen.1000788)
Supplement: Table S2 — Plasmids. (0.08 MB DOC) [file pgen.1000788.s009.doc]

**Table S2.** Plasmids

| Plasmid | Description | Citation |
| --- | --- | --- |
| pAL8 | pEB52 P*trc*-*mgrB*. AmpR | This work |
| pAL25 | pKT25 P*lac*-*cyaA*­­T25*::mgrB* KanR | This work |
| pAL27 | pKT25 P*lac-cyaA*­­T25*::phoQ* KanR | This work |
| pAL33 | pUT18C P*lac-cyaA*­­T18*::mgrB* AmpR | This work |
| pAL34 | pGFPmut3.1 P*lac-gfpA206K::mgrB* AmpR | This work |
| pAL36 | pKT25 P*lac-cyaA*­­T25*::phoQ*chim KanR | This work |
| pAL38 | pEB52 P*trc-gfpA206K::mgrB*. AmpR | This work |
| pAL39 | pEB52 P*trc-gfpA206K*. AmpR | This work |
| pAL41 | pUT18 P*lac*-*phoQ::cyaA*T18 AmpR | This work |
| pAL42 | pEB52 P*trc-mgrB* (*Y. pestis*). AmpR | This work |
| pAL43 | pEB52 P*trc-mgrB* (*S. enterica*). AmpR | This work |
| pAL46 | pUT18 P*lac*-*phoQ*chim*::cyaA*T18 AmpR | This work |
| pCP20 | *orits*, FLP recombinase expression plasmid. AmpR,CmR | [1] |
| pEB52 | pTrc99a with the NcoI site removed by cutting with NcoI, treating with mung bean nuclease, and re-ligating. AmpR | E. Batchelor and M. Goulian, unpublished |
| pGB2 | pSC101 derivative, SpcR | [2] |
| pGFPmut3.1 | *gfpmut3.1* | Clontech |
| pKT25 | *ori*p15A P­*lac*-*cyaA*T25-MCS KanR | Euromedex |
| pLPQ2 | pSC101  *phoPphoQ* driven by the *lacUV5* promoter. SpcR | [3] |
| pLPQ*2 | Derivative of pLPQ2, *phoQ* replaced by *phoQ*chim, which encodes a PhoQ hybrid with the periplasmic domain of *P. aeruginosa* PhoQ. SpcR | [4] |
| pMG68 | *gfpmut3.1*A206K AmpR | M. Goulian, unpublished |
| pNL2 | pGB2 P*phoN-lacZ* SpcR | [4] |
| pTM69 | pTrc99a Ptrc-*phoQ*, AmpR | T. Miyashiro and M. Goulian, unpublished |
| pTrc99a | *lacIq*, Ptrc-MCS, AmpR | [5] |
| pUT18 | pUC19 P*lac*-MCS-*cyaA*T18 AmpR | Euromedex |
| pUT18C | pUC19 P­*lac*-*cyaA*T18-MCS AmpR | Euromedex |

1. Cherepanov PP, Wackernagel W (1995) Gene disruption in Escherichia coli: TcR and KmR cassettes with the option of Flp-catalyzed excision of the antibiotic-resistance determinant. Gene 158: 9-14.

2. Churchward G, Belin D, Nagamine Y (1984) A pSC101-derived plasmid which shows no sequence homology to other commonly used cloning vectors. Gene 31: 165-171.

3. Waldburger CD, Sauer RT (1996) Signal detection by the PhoQ sensor-transmitter. Characterization of the sensor domain and a response-impaired mutant that identifies ligand-binding determinants. J Biol Chem 271: 26630-26636.

4. Lesley JA, Waldburger CD (2001) Comparison of the Pseudomonas aeruginosa and Escherichia coli PhoQ sensor domains: evidence for distinct mechanisms of signal detection. J Biol Chem 276: 30827-30833.

5. Amann E, Ochs B, Abel KJ (1988) Tightly regulated tac promoter vectors useful for the expression of unfused and fused proteins in Escherichia coli. Gene 69: 301-315.
