# Supplementary material for: Feedback Inhibition in the PhoQ/PhoP Signaling System by a Membrane Peptide
Source: PLoS Genet. 2009 Dec 24;5(12):e1000788. doi: 10.1371/journal.pgen.1000788 (PMC2789325; doi:10.1371/journal.pgen.1000788)
Supplement: Table S3 — Primers. (0.04 MB DOC) [file pgen.1000788.s010.doc]

**Table S3.** Primers

| Primer Name | Primer Sequence (5' to 3') |
| --- | --- |
| Bam-mgrB-1R | AAAGGATCCTCACCACGGGATAAACTGG |
| Eco-rbsMgrB-1F | GCTAGAATTCTGACATAAGGTAGGTG |
| GFP-mgrB-1F | GGCATGGATGAACTATACAAAATGAAAAAGTTTCGATGGG |
| GFP-mgrB-1R | CCCATCGAAACTTTTTCATTTTGTATAGTTCATCCATGCC |
| HindIII-phoQ-CTH-1F | AAAAAGCTTGATGAAAAAATTACTGCGTC |
| KpnI-CTH-mgrB-1R | TAAGGTACCCTACTTATTCACCACGGGATAAACTGG |
| KpnI-CTH-phoQ-1R | TAAGGTACCCTACTTATTTATTCATCTTTCGGCGC |
| pCAH63-u1 | ACTTTATGCTTCCGGCTCGT |
| Salmon-1F | CATGAATTCTAAGATCGGAGAGTGGA |
| Salmon-1R | GTAGGATCCTTACCAGGGAATAAATTTATTGATGG |
| Sal-PhoQ-CTH-1F | AAAAGTCGACATGAAAAAATTACTGCGTC |
| Spe-mgrB-1R | AAAACTAGTTCACCACGGGATAAACTGG |
| XbaI-CTH-mgrB-1F | ACATCTAGAGATGAAAAAGTTTCGATGGG |
| XbaI-CTH-phoQ-1F | ACATCTAGAGATGAAAAAATTACTGCGTC |
| XbaI-PhoQ-CTH-1R | TTTTCTAGAGTTTCATCTTTCGGCGCAGAATGC |
| Yellow-1F | TAAGAATTCTGATAAATAAGGGTGCTCG |
| Yellow-1R | GTTGGATCCTTACCAGGGAACAATCGTAGTAATAGCAC |
