# Supplementary material for: Feedback Inhibition in the PhoQ/PhoP Signaling System by a Membrane Peptide
Source: PLoS Genet. 2009 Dec 24;5(12):e1000788. doi: 10.1371/journal.pgen.1000788 (PMC2789325; doi:10.1371/journal.pgen.1000788)
Supplement: Text S1 — Plasmid construction. (0.04 MB DOC) [file pgen.1000788.s011.doc]

### Text S1

### Plasmid Construction

A table of primers used for the construct of plasmids is given in Table S3. All DNA segments that were amplified by PCR and cloned into plasmids were verified to be free of mutations by DNA sequencing.

The plasmid pAL8 contains *mgrB* in front of the *trc* promoter in a vector derived from pTrc99a [1]. To construct pAL8, the *mgrB* gene was amplified from *E. coli* genomic DNA using primers Eco-rbsMgrB-1F and Bam-mgrB-1R (Table S3), cut with EcoRI and BamHI and ligated to the EcoRI and BamHI sites in pEB52.

The plasmids pAL25, pAL27, and pAL36 are derivatives of pKT25 (Euromedex, Souffelweyersheim, France) that express N-terminal translational fusions of the T25 subunit of *Bordetella* adenylyl cyclase to MgrB, PhoQ, and PhoQ_chim_, respectively. To construct pAL25, *mgrB* was amplified from pAL8 using primers XbaI-CTH-mgrB-1F and KpnI-CTH-mgrB-1R , cut with XbaI and KpnI and ligated into the XbaI and KpnI sites of pKT25. The plasmid pAL27 was constructed by amplifying *phoQ* from pTM69 with primers XbaI-CTH-phoQ-1F and KpnI-CTH-phoQ-1R and ligating into the XbaI and KpnI sites of pKT25. The plasmid pAL36 was constructed in a similar fashion using primers XbaI-CTH-phoQ-1F and KpnI-CTH-phoQ-1R and pLPQ*2 [2] as the template for the chimeric *phoQ* construct.

The plasmid pAL33 is a derivative of pUT18C (Euromedex) that encodes an N-terminal fusion of the T18 subunit of *Bordetella* adenylyl cyclase to MgrB. To construct pAL33, *mgrB* was amplified from pAL8 with primers XbaI-CTH-mgrB-1F and KpnI-CTH-mgrB-1R, cut with XbaI and KpnI and ligated into the XbaI and KpnI sites of pUT18C.

The plasmids pAL41 and pAL46 are derivatives of pUT18 (Euromedex) that express fusions of the T18 subunit of Bordatella adenylyl cyclase to C-terminus of PhoQ and PhoQ_chim_, respectively. To construct pAL41, *phoQ* was amplified from pTM69 using primers HindIII-phoQ-CTH-1F and XbaI-PhoQ-CTH-1R, cut with HindIII and XbaI and ligated into the HindIII and XbaI sites of pUT18. The plasmid pAL46 was constructed by amplifying *phoQ_chim_* from pAL36 using primers Sal-PhoQ-CTH-1F and Xba-PhoQ-CTH-1R and inserting into the SalI and XbaI sites of pUT18.

The plasmid pAL38 expresses a fusion of GFP3.1A206K, a monomeric derivative of GFPmut3.1, to the N-terminus of MgrB. The plasmid was constructed by overlap-extension PCR using primers pCAH63-u1 and GFP-mgrB-1R with template pMG68, and primers GFP-mgrB-1F and Spe-mgrB-1R with template pAL8, respectively. The resulting PCR product was cut with SpeI and PstI and ligated in the SpeI and PstI sites of pGFPmut3.1 (Clontech, Mountain View, CA), which resulted in pAL34. This plasmid was then cut with XmaI and SpeI and the 893 bp fragment was ligated to the XmaI and XbaI sites of pEB52, to give pAL38. The plasmid pAL39 was constructed by subcloning the 769bp XmaI/SpeI fragment of pMG68 into the XmaI/XbaI sites of pEB52.

The plasmids pAL42 and pAL43 contain the *Y. pestis* KIM and *Salmonella enterica* subsp. enterica serovar Typhimurium *mgrB* homologs, respectively, cloned in front of the *trc* promoter. The *mgrB* gene from KIM6 genomic DNA was amplified with primers Yellow-1F and Yellow-1R, cut with EcoRI and BamHI and ligated to the EcoRI and BamHI sites of pEB52 to give pAL42. The plasmid pAL43 was constructed in a similar fashion using primers Salmon-1F and Salmon-1R and strain 14028s genomic DNA.

**References**

1. Amann E, Ochs B, Abel KJ (1988) Tightly regulated tac promoter vectors useful for the expression of unfused and fused proteins in Escherichia coli. Gene 69: 301-315.

2. Lesley JA, Waldburger CD (2001) Comparison of the Pseudomonas aeruginosa and Escherichia coli PhoQ sensor domains: evidence for distinct mechanisms of signal detection. J Biol Chem 276: 30827-30833.
